# Sustainable Performance Evaluation: Evidence from Listed Chinese Mining Corporations

**DOI:** 10.3390/e23030349

**Published:** 2021-03-15

**Authors:** Yuan Ma, Jingzhi Men, Mingyu Li, Xiaoyan Li

**Affiliations:** College of Economics and Management, Shandong University of Science and Technology, Qingdao 266590, China; may@sdust.edu.cn (Y.M.); mjzadeline@163.com (J.M.); 17854259419@163.com (M.L.)

**Keywords:** social performance, environmental performance, economic performance, responsibility management, entropy-weight-based TOPSIS method

## Abstract

Rapid industrial development has caused a series of environmental problems, which is not conducive to sustainable development of society as a whole. It is necessary to build a sustainable development evaluation system. Most of the existing literature has evaluated corporate sustainable performance from the economy, environment and society on the basis of triple bottom lines. Considering the research gap and the practice need, an evaluation system is established from four dimensions, referred to as economy, society, environment and responsibility management, and 29 indicators are designed to measure these four dimensions. Twenty seven listed Chinese mining corporations are selected as research samples, and the entropy-weight-based Technique for Order of Preference by Similarity to Ideal Solution (TOPSIS) method is applied to calculate indicators’ weights. Results show that the four dimensions of sustainable performance weights from high to low are society, environment, economy, and management process.

## 1. Introduction

Due to the environmental degradation caused by industrial development, sustainable development (SD) has become a major concern for countries, firms and individuals [1]. Some scholars believe that firms are the only organizations that have resources, technology, and motivation to achieve sustainability, so firms have a greater potential contribution to SD of countries and regions [2]. Considering the urgency of SD and the potential influence of firms, scholars have linked firms with SD and proposed the concept of corporate sustainable performance (CSP), which is used to represent the status and level of corporate SD [3,4].

A reasonable indicator system should be established to evaluate CSP. The development of performance indicators is conducive to optimizing operations, connecting and weighing all aspects of SD [5], in order to guide firms to take specific management actions to improve corporate sustainability [6,7,8].

Scholars have generally established CSP evaluation indicators based on triple bottom lines [9]. The most recognized is the Global Reporting Initiative (GRI) framework [10,11,12]. It developed a corporate sustainable assessment framework to guide firms in the preparation of corporate sustainability reports on environmental, social and economic impacts [5] for the following categories: economic, environmental, financial, employee and workplace related, product related and social related [10,11,12]. However, the biggest disadvantage of the GRI framework is that indicators are basically prepared for external reporting, and their impact on the actual management process of sustainable performance is limited [5].

The existing research on CSP evaluation has achieved valuable results, yet several deficiencies still exist. First, most studies adopt triple bottom lines to analyze CSP, with the emphasis on economy, environment, and society, lacking a consideration of firms’ management process [13,14,15,16]. International Standards Organization believes that natural environment may be influenced due to firms’ operation, which calls for the implementation of appropriate measures to reduce such impacts. Additionally, the concept of management performance index has been put forward in ISO14031. Therefore, it is imperative to add management indicators on the basis of the triple bottom lines. Second, most researches focusing on CSP are taking developed countries as samples, lacking an attention of developing countries [1,13,17,18]. However, unlike developed countries, the weakness of economic capacity in developing countries calls for vigorous improvement of their national strength. As is often the case, the pursuit of economic growth is bound to expose the countries to greater pressure for SD. Therefore, developing countries need to attach more attention to SD, especially, the establishment of evaluation indicators on CSP can guide firms to improve their sustainability, exploit their potential contribution in sustainability, thus promote national and regional SD.

The entropy-weight-based Technique for Order of Preference by Similarity to Ideal Solution (TOPSIS) method is adopted to construct the evaluation framework of CSP, and listed Chinese mining corporations are chosen as samples to verify the rationality and practicability of this evaluation framework. China put forward the strategy of SD in 1992 and paid more and more attention to SD in the past 10 years, however, energy production and consumption had increased year by year due to the vigorous economic development since the reform and opening up. Mining firms bear the heavy responsibility in SD. Therefore, it is suitable to select Chinese mining firms as elements of a to-be-studied sample, which will not only recover the shortcomings of existing research but also make the samples more representative.

The contributions of this paper are as follows. First, the responsibility management index is added to the evaluation index of CSP, which focuses on firms’ management process. Although scholars have tried to establish the evaluation indicators of CSP [5,9,10,11,12,13], most of them have focused on economic performance, social performance and environmental performance, ignoring corporate management process [19,20,21,22]. Second, mining firms are selected as elements of a to-be-studied sample. Existing research on sustainability assessment mainly comes from three views, from the regional development view [15,23,24], from the supply chain view [19,20,25], and from the industry view, such as manufacturing industry [1], food and beverage industry [7], electronics industry [18], furniture industry [21], metallurgical industry [22], chemical industry [26], banking industry [27]. Research on mining industry is still scarce and far from meeting the needs of real businesses. At one extreme, it generates wealth from the land, and at the other extreme, it disrupts the environment and the community life around it [24]. Almost all operations in the mining industry are related to environmental protection, social responsibility and economic benefits. Considering the research gap mining firms are selected.

Overall, we find that the four dimensions of sustainable performance with the weight from high to low are society, environment, economy and management, indicating that social performance occupies the most significant role in the SD of mining firms, followed by the environment performance. The conclusions can provide practical implications for the sustainable development of mining firms. 

## 2. Literature Review

The World Commission on Environment and Development first proposed the concept of SD in 1987, arguing that SD is the fulfillment of the needs of the present day without harming future generations in meeting their own needs [28]. With the advancement of times and academic exploration, the concept of SD is gradually extended into the process of corporate development. Scholars employ CSP to denote the outcomes of corporate sustainability. Moreover, corporate sustainable development and relating performance measures frequently consider economic, environmental, and social issues. While ensuring economic development and accountability to shareholders, firms also need to meet the needs of direct and indirect stakeholders (such as employees, customers, communities, etc.) [29]. 

The term Triple Bottom Lines was first coined by Elkington [30]. Some scholars believe that measuring CSP based on the triple bottom lines allows evaluating whether a firm meets the criteria for sustainability from three aspects of economy, environment and society [19,20]. On the basis of the triple bottom lines, the GRI, co-issued by the US non-profit environmental economic organization and the United Nations Environment Program, established a framework for CSR reporting. The GRI provides a reference for academia to measure CSP [10,11,12]. Drawing on the experience of GRI, Rankins Corporate Social Responsibility Ratings (RKS) has been issued by Chinese corporate responsibility third party rating agency since 2009.

Although GRI is widely recognized, scholars have not stopped their efforts to rebuild the sustainable performance evaluation framework for firms. Academia hopes to guide firms’ SD more scientifically through more sample research. Lee and Saen [18] evaluate the inputs and outputs of Korean electronic manufacturing firms from 3 aspects, economic profitability, social responsibility, and environmental sustainability. Considering the impact of firm size on CSP, Cagno et al. [17] set up indicators for 3 different sizes of firms. Aras et al. [31] add corporate governance into the triple bottom lines to evaluate CSP. Specially, Tan et al. [1] establish an indicator framework using a systematic indicator selection method in order to facilitate the SD of small and medium sized firms in Singapore. Besides the triple bottom lines that are often mentioned in extant research, performance management is added in his framework, and 9 sub-indicators are identified. Although management indicator is included in Tan et al. [1], there are still lack of discussion and demonstration of indicator weight, which limits the guidance of the research conclusion to the SD of firms. 

While constructing evaluation indicators of CSP, models to measure it based on different methods have been proposed [21,22,32]. Commonly used methods include Delphi method [25], analytic hierarchy process [26,33], data envelopment analysis [18,23], fuzzy logic-based approach [24], fuzzy PROMETHEE II method [20], fuzzy TOPSIS [19,31], decision tree modeling approach [15] and life-cycle inventory method [34].

## 3. Corporate Sustainable Performance Index System

Based on the triple bottom lines [30], which aims to measure the economic, social, and environmental performance of a company over time, and considering the management process, the CSP evaluation index is constructed in terms of economic performance, environmental performance, social performance, and responsibility management. 

### 3.1. Economic Performance

Economic performance reflects the value of firms’ existence and is an important aspect of CSP, because profit can maintain firms’ survival and support their development [9]. Under the context of SD, firms’ economic sustainability should ensure long-term effective profitability of corporate financial capital [35], because economically stable firms can guarantee cash flow to meet capital liquidity at any time, and provide stakeholders with continuous returns above the average level [36], so as to ensure themselves keep on sustainability.

Firms’ economic performance indicators are evaluated from three aspects: financial performance, solvency, and firm growth. Financial performance provides information on the return of firms’ funds, solvency guarantees the safety of firms’ assets, and firm growth is the source of SD.

### 3.2. Environmental Performance

Environmental performance relates to the impact of an organization on biological and non-biological natural systems, including ecosystems, land, air, and water. The GRI has revealed several indicators of environmental performance, such as materials, energy, water, biodiversity, emissions, and compliance [10,11]. Studies have also shown that environmental performance should disclose such aspects as environmental policy, organizational responsibility, training and awareness, monitoring and follow-up activities [37]. Relative evaluations of environmental performance have been conducted by different organizations and scholars, so that such evaluations can operate in various social, economic, and environmental contexts to serve different purposes [38]. Firms’ environmental performance indicators are constructed of environmental initiatives, energy conservation, as well as pollution abatement and emission reduction.

### 3.3. Social Performance

Social performance is closely related to moral norms. Firms are responsible for producing and selling goods, as well as providing services to benefit from the whole society. Therefore, each firm is obliged to feedback the supports of community and stakeholders [39] to create value for the public and society affected by it [9]. Social performance usually focuses on issues related to internal business environment, such as human resource practices and employee health and safety [40], as well as issues related to stakeholders and the public in external business environment [41]. In addition, social performance also requires firms to comply with laws and regulations, concern human rights of employees, ensure product quality, and pay attention to regional philanthropy [9,39]. In summary, we argue that firms should be responsible for the laws, employees, products, and community, based on which, relevant indicators of social performance are proposed.

### 3.4. Responsibility Management

On the basis of the triple bottom line, the importance of management process to firms’ sustainable performance is fully taken into consideration, therefore, the dimension of responsibility management is added to the sustainable performance evaluation. Responsibility management is employed to measure the formulation and adoption of management measures and weigh up the management performance in terms of firms’ SD. This dimension mainly focuses on the efforts of firms in setting responsibility concept, establishing responsible organization, formulating responsibility strategy and promoting responsibility integration. Responsibility management is not easily monitored and is seldom involved in studies of sustainable performance measurement [42]. However, appropriate sustainable management measures play a vital role in obtaining favorable sustainable performance. As such, it is necessary to formulate and implement management measures that can ensure firms’ performance level to realize sustainable goals [5]. It is indicated that management indicators are another pillar of traditional sustainability, which can be used to measure firms’ management performance in SD [1]. Neugebauer, et al. [43] also believe that the severe SD pressure increases the necessity to develop SD management initiatives, which is conducive to promote and guide firms’ sustainable practices. In addition, firms need to employ strategy development and rigorous management to meet stakeholders’ requirements to achieve sustainable development. In summary, the index framework of this paper includes four dimensions and 29 indicators, and the specific indicators are shown in Table 1.

## 4. Evaluation Methods and Data Sources

### 4.1. The Entropy-Weight-Based TOPSIS Method

The entropy-weight-based TOPSIS method is a modification of the traditional TOPSIS evaluation method, by first determining the weights of the evaluation indexes through the entropy weight method, and then determining the ranking of the evaluation objects through the TOPSIS method using approximate the ideal solution.

The entropy-weight method objectively determines the weight according to the information provided by each evaluation indicator to be entropy weight. It can objectively reflect the importance of a certain indicator in the indicator system and has strong fitness during weight determination [27].

TOPSIS is a multi-criteria decision method, developed by Hwang and Yoon [44]. Its core ideology is to calculate the distances between the evaluation objects and the best (worst) solutions, based on which, the relative closeness can be calculated, and each scheme can be ranked [31,45].

### 4.2. Detailed Steps

#### 4.2.1. Calculating the Index Weights

The weights of each index are calculated by the entropy weight method, and the specific calculation steps are as follows:

(1) Constructing original information matrix;
  $$X={\left(X_{ij}\right)}_{np}\;X=\left(\begin{array}{cccc} X_{11} & X_{12} & \ldots & X_{1p}\\ X_{21} & X_{22} & \ldots & X_{2p}\\ \ldots & \ldots & \ldots & \ldots \\ X_{n1} & X_{n2} & \ldots & X_{np} \end{array}\right)$$
(2) Standardized processing;
  $R={\left(r_{ij}\right)}_{n\times p}$ is obtained by normalizing the *X*, in which *i =* 1, …, *n*; *j =* 1, …, *p*; $r_{ij}\in [0,1]$.
  In addition, different meanings are in the positive index and negative index values (the positive index value is the higher the better, but the reverse is negative index), thus heterogeneous algorithms for data standardization of positive and negative indexes are performed.
  The positive index:
  (1) $$r_{ij}=\frac{X_{ij}-\min X_{ij}}{\max X_{ij}-\min X_{ij}}$$
  The negative index:
  (2) $$r_{ij}=\frac{\max X_{ij}-X_{ij}}{\max X_{ij}-\min X_{ij}}$$
  The moderate index:
  (3) $$r_{ij}=1-\frac{\left|X_{ij}-\bar{X_{ij}}\right|}{\max\left|X_{ij}-\bar{X_{ij}}\right|}$$
(3) Calculating the proportion of evaluation object *i* under indicator *j*;
  (4) $$P_{ij}=\frac{r_{ij}}{\sum_{i=1}^{n}r_{ij}}$$
(4) Calculating the entropy of the *j*th index;
  (5) $$e_{j}=-k\sum_{i=1}^{n}P_{ij}\ln P_{ij}$$
  In which $k=\frac{1}{\ln n}$, and hypotheses when $P_{ij}=0$, $P_{ij}\ln P_{ij}=0$.
(5) Calculating the entropy weight of the *j*th index
  (6) $$w_{j}=\frac{1-e_{j}}{\sum_{j=1}^{p}\left(1-e_{j}\right)}$$

#### 4.2.2. Constructing Evaluation Model

The specific steps of TOPSIS method are as follows:

1. Transform original matrix X into standard normalized matrix $Y={\left(Y_{ij}\right)}_{np}$, in which *i =* 1, …, *n*; *j =* 1, …, *p*.
  (7) $$Y_{i}{}_{j}=\frac{X_{ij}}{\sum_{i=1}^{n}X_{ij}}$$
2. Construct weighted normalization matrix $Z={\left(Z_{ij}\right)}_{np}$ according to the weight determined by Formula (6), in which is a standard normalized matrix based on Formula (7)
  (8) $$Z_{ij}=w_{j}Y_{ij}$$
3. Determine set $Z^{+}$ of positive ideal solutions and set $Z^{-}$ of negative ideal solutions
  (9) $$Z^{+}=\left\{\max Z_{ij}\left|i=1,2,\ldots ,n\right.\right\}$$
  (10) $$Z^{-}=\left\{\min Z_{ij}\left|i=1,2,\ldots ,n\right.\right\}$$
4. Calculating Euclidean distance between evaluation objects and positive (negative) ideal solutions:
  (11) $$D^{+}=\sqrt{\sum_{j=1}^{p}{\left(Z_{ij}^{+}-Z_{ij}\right)}^{2}}$$
  (12) $$D^{-}=\sqrt{\sum_{j=1}^{p}{\left(Z_{ij}^{-}-Z_{ij}\right)}^{2}}$$
5. Calculating comprehensive evaluation index of evaluation objects:
  (13) $$C_{i}=\frac{D_{i}^{-}}{D_{i}^{-}+D_{i}^{+}}$$

### 4.3. Sample Selection and Data Source

In this paper, we amassed data of listed mining corporations from corporate financial reports and corporate social responsibility reports published in 2018 and 2019 from the China Stock Market & Accounting Research (CSMAR) database, the web of giant tide and corporate official network. After removing firms with incomplete information, 27 corporations were left as the samples of this paper. The descriptive statistics of these indicators are depicted by Table A1 in Appendix A.

## 5. Analysis

### 5.1. Calculation of Evaluation Index Value

According to the above indicator framework in Table 1, the entropy weight of indicators needs to be defined firstly. The indicators selected in this paper include several positive indicators, whose optimal solution is the maximum value and the worst solution is the minimum value, and vice versa if there are negative indicators. In addition, several moderate indexes are also selected, including return on investment, short-term solvency, long-term solvency, reasonable compensation, and the proportion of female executives. Since the values of sustainable development concept, institutional sector and compliance system construction are 1, their entropy weights are 0.

### 5.2. Standardization and Determination of Entropy Weight

Due to the nonuniformity of measurement units of each index, standardized processing is indispensable before calculating the comprehensive index with the index data, that is, the heterogeneous indexes need to be homogenized first. The method in Formulas (1)–(3) is used in the standardized process based on the nature of index (positive index, negative index or moderate index). Following steps of the entropy weight method, the proportion of the *i*th evaluation object in the *j*th index, the entropy value of the *j*th index, and the entropy weight value of the *j*th index are calculated according to the formula. Then the index is sorted out according to the dimension it belongs to, and the entropy weight value of the comprehensive index is obtained. The specific calculation results are shown in Table 2.

According to Table 2, excluding the indicators of sustainable development concept, institutional sector and compliance system construction, the weight difference of the remaining 26 indicators is not obvious, indicating minor differences in these indicators among selected firms, and almost the same individual impact degree for sustainable performance score. However, after sorting out and calculating the indicators according to the dimensions they belong to, it can be seen that the weights of environmental performance and social performance are significantly higher than the weights of responsibility management and economic performance, which are 34.1961% and 39.0993%, respectively, playing a vital role in corporate sustainable performance. The weight of economic performance is 19.5590%, moderately influencing corporate sustainable performance. While the weight of responsibility management is 7.1457%, contributing slightly to corporate sustainable performance.

### 5.3. Results of TOPSIS Comprehensive Evaluation

After calculating the weight of each indicator by the entropy weight method, the comprehensive evaluation of corporate sustainable performance is conducted by the TOPSIS method. The ranking results of sample analysis are shown in Table 3.

The score of comprehensive evaluation value *Ci* obtained by TOPSIS method is distributed in the range of 0 to 1. The closer the score is to 1, the higher the CSP is, on the contrary, the closer the score is to 0, the worse the CSP is. It can be seen from Table 3 that the comprehensive evaluation values of the top five firms with sustainable performance are 0.5482, 0.4517, 0.4382, 0.4265 and 0.4050, respectively, indicating better sustainable performance. However, the bottom five firms’ are 0.2748, 0.2619, 0.2578, 0.2510 and 0.2130, respectively, indicating poor sustainable performance, which calls for further improvement.

The evaluation value and ranking of the top five firms in terms of economic performance, environmental performance, social performance and responsibility management are listed in Table 4. It can be seen from Table 4 that a minor differentiation in economic performance exists among those four firms except for Jizhong Energy Group (000937) ranked the 18th, and a relatively separated distribution exists among those five firms in terms of environmental performance, ranking from the second to the 26th. Moreover, only Hainan Mining (601969) ranks poorly in terms of social performance. Additionally, although the five firms score near the top in responsibility management, there are large deviations in the evaluation results. Finally, according to the results of Table 4, it is indicated that the poor rankings in environmental performance and social performance of Hainan Mining (601969) and Shaanxi Coal Industry (601225) result in their lower comprehensive rankings in Table 3.

### 5.4. Result Discussion

Most studies have classified CSP into economic performance, environmental performance and social performance, and affirmed the significance of environmental and social performance in the process of corporate sustainable development [13,17]. This paper also gives positive comments about the environmental and social performance in CSP evaluation. However, after comparing and sorting the existing literature, the triple bottom lines is limited obviously in that it only considers the economy, environment and society so that inevitably lacks attention of corporate management process. In view of this limitation, the management performance indicator, referred to as responsibility management, is added to the evaluation system. Although the comprehensive evaluation value of responsible management is the lowest among the four dimensions, it also indicates to some extent that the corporate management process contributes to the CSP.

Although there is relatively mature sustainability evaluation institutions in China, such as RKS, the samples it uses are 800 constituent corporations that are listed in the capital market and lack of special attention to a certain industry. Considering the differences between industries in SD, we chose a single industry. Therefore, in terms of evaluation results, it is difficult to find comparable conclusions in existing studies. To further highlight the characteristics and significance of the results, we comparatively analyze the research of Aras et al. [31] which similarly explored the sustainable performance of Turkish banking sector based on triple bottom lines with the entropy-weight TOPSIS method. It divided CSP into four dimensions of economic performance, environmental performance, social performance and corporate governance, and indicated the entropy weight of economic performance is the highest, followed by environmental performance, social performance and corporate governance. While our research classifies CSP into four dimensions and the entropy weight of those four dimensions from high to low is social performance, environmental performance, economic performance and responsibility management. It is indicated that the degree of urgency in terms of economy, society and environment is different when facing the problem of sustainable development. For the mining industry, social performance related to safety and employee benefits and environmental problems caused by mining influence sustainable development much more compared with economic performance.

Listed mining corporations are selected as research samples. Their strong economic strength leads to minor differences in terms of economic performance, but a large difference exists in environmental performance and social performance, resulting in higher weight results. It is indicated that owing to China’ s rapid economic growth over the past 20 years, some large firms have accumulated strong economic foundation, therefore, their competition for sustainable performance mainly focuses on social performance and environmental performance. The Chinese government has realized the necessity of SD. In the past decade, several norms on SD have been formulated and constantly improved and perfected. Furthermore, the concept of green development and sustainable development has been proposed in the 12th Five-Year Plan and the 13th Five-Year Plan. With increasingly improved awareness of protecting the environment and contributing to society and ever-growing concerns about the environment and society, Chinese firms have gradually realized that economic development is not the only purpose, but the ecological environment and social support are development foundations. Therefore, great efforts have been put into environment and society. Specifically, the high weight results of environmental and social performance in this paper are the evidence showing emphasis that firms paid to the environment and society.

In order to further analyze the comprehensive evaluation results of CSP and provide inspirations for other firms to improve their sustainable performance, this paper analyzes the social responsibility reports disclosed by these five firms in detail and tracks their public information. These firms have carried out comprehensive work in protecting the environment and contributing to society, for example, they have generally established an environmental management system, and have constantly tried to develop new production mode of energy conservation as well as adopted environmental protection measures to reduce pollution emissions. Taking China Molybdenum (603993) as an example, it updated the environmental policy applicable to the whole group, discussing various issues such as tailings management, closure plan and reclamation, biodiversity, energy, greenhouse gas emissions and air quality in February 2020. The mining areas operated by China Molybdenum have been equipped with mature environmental management system (EMS) and passed the ISO14001 certification. Specifically, China Molybdenum has formulated standards in tailings management with six key elements of governance, including (1) accountability, responsibility and ability, (2) planning and resource allocation, (3) risk management, (4) change management, (5) emergency preparation and response, (6) audit and verification. China Molybdenum also have endeavored to protect environmental and conserve resources. For example, it is committed to maximizing resources utilization, reducing waste generation, using circulating water in production, and continuously improving water saving technology, as well as protecting underground water, which helps saving much water resources. China Molybdenum has repeatedly changed its energy structure by using renewable energy while increasing input of new energy, in addition, strict control of pollutant emissions has been implemented to reduce the environmental impact of exhaust gas and waste emissions as far as possible. China Molybdenum has attracted a large number of professional talents, giving priority to employees’ safe and health through regularly employee training and constantly improvement of safety measures, for example. In addition to the above, China Molybdenum has so far been concerned with social and community development, and strongly supported education. In 2019, it contributed about CHY 2.67 million to the education cause of Luanchuan County, and provided material assistance to students. Moreover, it provided animal husbandry training and assistance to 267 farmers from 14 local villages, and cumulatively contributed about CHY 60 million to the public housing project in Luanchuan county. For lack of space, the evaluation values and rankings of the bottom 5 firms do not be listed in detail. By further comparing their financial reports and corporate social responsibility reports, it is found that several problems do exist in these firms such as lower management level, inefficient environmental management, less implementation of environmental protection measures, aging and infrequent maintenance of environmental protection facilities, and less voluntary activities.

## 6. Conclusions

This paper proposes a new framework for sustainable performance evaluation considering the dimensions of economy, environment, society, and management. The entropy-weight-based TOPSIS method is used to calculate the weight and listed Chinese mining firms are selected as samples for quantitative analysis. The four dimensions of sustainable performance with the weight from high to low are society, environment, economy and management.

The conclusions can provide several implications for both academia and practitioners. Theoretically, when studying corporate SD, scholars should not only focus on the triple bottom line and other results, but also take the management process into consideration. The existence of appropriate sustainable management measures to a certain extent determines whether firms can obtain good sustainable performance. Although the management process of SD is not easy to be monitored, it is necessary for academia to consider its role in the overall management of corporate sustainable development. The research on the management process of corporate sustainable development is to study sustainability at a higher corporate management hierarchy and guide the sustainability. Firms with SD concepts and appropriate SD management initiatives are more likely to achieve higher sustainability performance [5]. Therefore, the corporate management process or other relevant factors should be taken into account apart from the considerations of economy, environment and society when studying CSP, which is beneficial to improve corporate management process thus contribute to firms’ SD.

This study also provides implications for corporate managers. Corporate managers should change the original concept only focusing on economic performance, because SD is an important manifestation of firms’ future competitiveness. Efforts that should be put into the environment and society cannot be ignored, furthermore, firms should pay more attention to environmental and social performance while focusing on economic performance. Firms with poor sustainable performance can draw from those with good sustainable performance, such as constant adjustment of corporate SD management system, adoption of environmental-friendly supporting equipment in the process of product production, implementation of green innovation to reduce environmental pollution, care and protection of employee rights, and support of social public welfare. In addition, responsibility management also has a great contribution to improving CSP. Firms can establish a SD department to coordinate their SD management and promote their sustainability by means of carrying out SD training, enhancing the sustainable awareness of departments, and formulating reasonable management plans.

It is also worth reminding that our research has suffered from some limitations. First, fewer research samples are available because a relatively authoritative SD system has not been established, together with firms’ unmatured awareness of undertaking social responsibility and varying disclosure quality of SD. Second, only a single industry is selected as the research sample in this paper, lacking a consideration of other industry characteristics. Therefore, research of SD of more industries is encouraged and more detailed explorations of CSP should be conducted in the future, including evaluation models, evaluation frameworks, and even the discovery of theoretical bases.

## Figures and Tables

**Table 1:** Corporate sustainable performance index framework.

Dimensions	First-Grade Index	Second-Grade Index	Descriptions
Economic performance	Financial performance	Net profit margin on sales	P01 Net profit/sales revenue, positive index
		return on investment	P02 Dividend distribution rate = dividend per share/earnings per share, moderate index
	Solvency	short-term solvency	P03 Quick ratio = (current assets − inventory)/current liabilities, moderate index
		long-term solvency	P04 Asset-liability ratio = total liabilities/total assets, moderate index
	Enterprise growth	Growth rate of main business revenue	P05 (Current main business income − previous main business income)/previous main business income, positive index
Environmental performance	Environmental initiatives	Environmental management system	P06 Whether to establish an environmental management system, yes = 1, no = 0
		Environmental training	P07 Whether to implement environmental awareness education and training annually, yes = 1, no = 0
		Environmental regulation	P08 The negative of the proportion of environmental protection tax to current operating revenue
	Energy conservation	Energy conservation measures	P09 Whether to formulate energy conservation measures, yes = 1, no = 0
		Resource conservation measures	P10 Whether to formulate resource conservation measures, yes = 1, no = 0
		Usage of renewable resources	P11 Whether to use renewable resources as much as possible, yes = 1, no = 0
		Resource recycling	P12 Whether resources can be recycled, yes = 1, no = 0
	Pollution abatement and emission reduction	Waste and pollutant emission control and measures	P13 Whether there are perfect measures of waste gas and wastewater discharge management, yes = 1, no = 0
		Waste utilization	P14 Whether wastes are comprehensively utilized, yes = 1, no = 0
Social performance	Compliance with laws	Compliance system construction	P15 Whether to comply with local government laws and regulations and conduct legal compliance training, yes = 1, no = 0
		Active tax payment	P16 Tax expense ratio = (various taxes paid − tax refunds received)/operating income
	Protection of employees	Reasonable compensation	P17 Employee remuneration rate = cash paid to and for employees/operating income, moderate index
		Safety and health	P18 Whether there is safety inspection and safety training, yes = 1, no = 0
		Fairness	P19 The proportion of female executives = number of women in senior management/total number of senior management, moderate index
		Communication channel	P20 Whether to establish a good internal communication mechanism with employees, yes = 1, no = 0
	Product production	Safety production management	P21 Whether to formulate safety production management system and conduct safety production training, yes = 1, no = 0
		Product quality control	P22 Whether there is strict product quality control, yes = 1, no = 0
	Community responsibility	Localization policy	P23 Whether participated in local activities this year, yes = 1, no = 0
		Community donation ratio	P24 Donation expenses/operating income, positive index
		Volunteer activities	P25 Whether there are measures to support volunteer activities, yes = 1, no = 0
Responsibility management	Notion of responsibility	Sustainable development concept	P26 Whether sustainable development is included in corporate philosophy, yes = 1, no = 0
	Responsible organization	Institutional sector	P27 Whether to establish a leading body and relevant organizational departments for firm’s sustainable work, yes = 1, no = 0
	Responsible strategy	Planning issues	P28 Whether to formulate strategic responsibility issues and plan responsibility for sustainable development, yes = 1, no = 0
	Responsibility integration	Work development	P29 Whether to promote sustainable responsibility performance management to work practice, yes = 1, no = 0

**Table 2:** Index entropy weights.

Dimensions	First-Grade Index	Second-Grade Index	Entropy Weights (%)	Comprehensive Entropy Weight (%)
Economic performance	Financial performance	Net profit margin on sales	3.9506	19.5590
		Investment return	3.9557	
	Solvency	Short-term solvency	3.9812	
		Long-term solvency	3.9268	
	Enterprise growth	Growth rate of main business revenue	3.7447	
Environmental performance	Environmental initiatives	Environmental management system	3.3511	34.1961
		Environmental training	3.5146	
	Energy conservation	Environmental regulation	3.9728	
		Energy conservation measures	3.9499	
		Resource conservation measures	3.8745	
		Usage of renewable resources	3.9064	
		Resource recycling	3.8791	
	Pollution abatement and emission reduction	Waste and pollutant emission control and measures	3.9009	
		Waste utilization	3.8468	
Social performance	Compliance with laws	Compliance system construction	0.0000	39.0993
		Active tax payment	4.0023	
	Protection of employees	Reasonable compensation	3.9339	
		Safety and health	4.1060	
		Fairness	3.9535	
		Communication channel	3.8854	
	Product production	Safety production management	3.9661	
		Product quality control	3.9406	
	Community responsibility	Localization policy	3.5959	
		Community donation ratio	3.7382	
		Volunteer activities	3.9774	
Responsibility management	Notion of responsibility	Sustainable development concept	0.0000	7.1457
	Responsible organization	Institutional sector	0.0000	
	Responsible strategy	Planning issues	3.2743	
	Responsibility integration	Work development	3.8713	

**Table 3:** Positive and negative ideal solution, comprehensive evaluation value and ranking.

Corporate Code	*D^+^*	*D^−^*	*C_i_*	Ranking	Corporate Code	*D^+^*	*D^−^*	*C_i_*	Ranking
603993	0.0146	0.0178	0.5482	1	601101	0.0198	0.0098	0.3308	15
000937	0.0184	0.0151	0.4517	2	600188	0.0192	0.0094	0.3292	16
601958	0.0179	0.0139	0.4382	3	600489	0.0199	0.0096	0.3267	17
601969	0.0171	0.0127	0.4265	4	600497	0.0202	0.0093	0.3149	18
601225	0.0177	0.0120	0.4050	5	000968	0.0218	0.0100	0.3142	19
601699	0.0184	0.0123	0.4000	6	601918	0.0212	0.0085	0.2868	20
600256	0.0191	0.0117	0.3806	7	600547	0.0210	0.0084	0.2852	21
600508	0.0180	0.0106	0.3701	8	601666	0.0202	0.0079	0.2817	22
601898	0.0184	0.0106	0.3648	9	600985	0.0209	0.0079	0.2748	23
000655	0.0195	0.0109	0.3586	10	002155	0.0207	0.0073	0.2619	24
600123	0.0198	0.0110	0.3569	11	600711	0.0208	0.0072	0.2578	25
600777	0.0196	0.0106	0.3516	12	000758	0.0226	0.0076	0.2510	26
000983	0.0192	0.0099	0.3407	13	601168	0.0216	0.0058	0.2130	27
601088	0.0203	0.0104	0.3394	14					

**Table 4:** Evaluation value and ranking of corporate sustainable performance.

Corporate Code	EcP	Ranking	EnP	Ranking	SP	Ranking	RM	Ranking
603993	0.5857	1	0.4386	9	0.4966	3	1.0000	1
000937	0.2203	18	0.7213	2	0.6300	2	0.2745	3
601958	0.4596	3	0.3549	15	0.4616	4	0.2745	3
601969	0.5797	2	0.1998	26	0.2322	26	0.2745	3
601225	0.3635	5	0.4386	9	0.3635	10	1.0000	1

EcP: Economic performance; EnP: Environmental performance; SP: Social performance; RM: Responsibility management.

## Data Availability

Not applicable.

## Appendix A

**Table A1:** Descriptive statistics.

Indicators	Observers	Min	Max	Mean	SD	Var.	Skewness	Kurtosis
Net profit margin on sales	27	−0.1050	0.2846	0.0762	0.0771	0.0059	0.8275	2.1170
return on investment	27	0	1.6908	0.3011	0.3471	0.1205	2.7018	9.5467
short-term solvency	27	0.1573	4.9935	1.1480	1.1591	1.3435	2.7957	7.7404
long-term solvency	27	0.1032	0.7558	0.5078	0.1705	0.0291	−0.9734	0.5086
Growth rate of main business revenue	27	−0.2516	1.6816	0.1879	0.4468	0.1996	2.8795	8.2586
Environmental management system	27	0	1	0.3333	0.4804	0.2308	0.7494	−1.5600
Environmental training	27	0	1	0.6667	0.4804	0.2308	−0.7494	−1.5600
Environmental regulation	27	−0.0009	0	−0.0002	0.0003	0	−1.5846	1.2980
Energy conservation measures	27	0	1	0.8519	0.3620	0.1311	−2.0994	2.5943
Resource conservation measures	27	0	1	0.8148	0.3958	0.1567	−1.7178	1.0211
Usage of renewable resources	27	0	1	0.7778	0.4237	0.1795	−1.4162	0
Resource recycling	27	0	1	0.7778	0.4237	0.1795	−1.4162	0
Waste and pollutant emission control and measures	27	0	1	0.9259	0.2669	0.0712	−3.4472	10.6704
Waste utilization	27	0	1	0.8519	0.3620	0.1311	−2.0994	2.5943
Compliance system construction	27	1	1	1	0	0		
Active tax payment	27	0.0105	0.2235	0.1011	0.0661	0.0044	0.3229	−1.1543
Reasonable compensation	27	0.0086	0.3634	0.1280	0.0940	0.0088	0.9196	−0.0727
Safety and health	27	1	1	1	0	0		
Fairness	27	0	0.2900	0.0607	0.0946	0.0090	1.2486	0.2170
Communication channel	27	0	1	0.8148	0.3958	0.1567	−1.7178	1.0211
Safety production management	27	0	1	0.9630	0.1925	0.0370	−5.1962	27.0000
Product quality control	27	0	1	0.8889	0.3203	0.1026	−2.6229	5.2650
Localization policy	27	0	1	0.7407	0.4466	0.1994	−1.1644	−0.7020
Community donation ratio	27	0	0.0015	0.0003	0.0004	0	1.8481	2.7536
Volunteer activities	27	0	1	0.8889	0.3203	0.1026	−2.6229	5.2650
Sustainable development concept	27	1	1	1	0	0		
Institutional sector	27	1	1	1	0	0		
Planning issues	27	0	1	0.2963	0.4653	0.2165	0.9456	−1.2008
Work development	27	0	1	0.9259	0.2669	0.0712	−3.4472	10.6704
